# Risk of Thrombosis during and after a SARS-CoV-2 Infection: Pathogenesis, Diagnostic Approach, and Management

**DOI:** 10.3390/hematolrep15020024

**Published:** 2023-04-03

**Authors:** Henry Sutanto, Gatot Soegiarto

**Affiliations:** 1 Department of Internal Medicine, Faculty of Medicine, Universitas Airlangga, Surabaya 60132, Indonesia; henry1988md@gmail.com; 2 Department of Internal Medicine, Dr. Soetomo Teaching Hospital, Surabaya 60286, Indonesia; 3 Division of Allergy and Clinical Immunology, Department of Internal Medicine, Faculty of Medicine, Universitas Airlangga, Surabaya 60132, Indonesia

**Keywords:** COVID-19, coronavirus, thromboembolism, coagulation, deep-vein thrombosis, pulmonary embolism

## Abstract

Coronavirus disease 2019 (COVID-19) increases the risk of thromboembolic events, especially in patients with severe infections requiring intensive care and cardiorespiratory support. COVID-19 patients with thromboembolic complications have a higher risk of death, and if they survive, these complications are expected to negatively affect these patients’ quality of life. Moreover, recent data reported that the risk of thromboembolism remains high months after a COVID-19 infection. Therefore, understanding the pathogenesis of thrombosis in the setting of COVID-19 may facilitate the early prevention and treatment of COVID-19-associated thromboembolism to reduce concomitant morbidity, mortality, and disability. This review will first discuss the clinical characteristics of COVID-19 infections, particularly with regard to the underlying pathophysiology. Then, the pathogenesis of COVID-19-associated thrombosis at the molecular and cellular levels will be comprehensively reviewed. Next, the clinical manifestations of venous and arterial thromboembolism in COVID-19 as well as the potential benefits of several laboratory markers of thrombosis will be further discussed. Lastly, the preventive and therapeutic management of thromboembolism during and after COVID-19 will also be explained.

## 1. Introduction

Coronavirus disease 2019 (COVID-19) is a disease caused by infection with severe acute respiratory syndrome coronavirus type 2 (SARS-CoV-2). This disease mainly attacks the respiratory tract but generally has multiorgan impacts with varying intensities, ranging from mild to severe, and it can even be life-threatening. Since the initial appearance of this disease in Wuhan, China, in December 2019, COVID-19 has infected more than 650 million people and has caused the death of more than 6.5 million people worldwide [1]. Septic shock and multiorgan failure resulting from a suppurative lung infection are the main direct causes of death [2]. In addition, in patients who died from COVID-19, there was also evidence of a history of thromboembolism. For instance, in a small autopsy study conducted in Germany, 23.1% of patients had pulmonary embolism (PE), and 7.7% had deep-vein thrombosis (DVT); meanwhile, another study reported that the incidence of DVT was as high as 58%, and severe PE was the direct cause of death in one-third of the autopsied patients [2,3].

Thromboembolism is one of the important clinical manifestations of COVID-19 and is a factor that can affect morbidity and mortality in COVID-19. Worldwide, the reported incidence of thromboembolism in COVID-19 infections varies from one study to another [4]. Interestingly, a compilation of data from various previous studies showed that the incidence of thromboembolism increases sharply in patients with a severe COVID-19 infection who require intensive care and cardiorespiratory support. In contrast, the rate of complications of arterial and venous thrombosis in COVID-19 was low in individuals with an asymptomatic or mild disease who were not hospitalized [4,5]. A meta-analysis of 102 studies involving 60,503 patients also reported the incidence of venous and arterial thromboembolism related to COVID-19, reaching 14.7% and 3.9%, respectively. Furthermore, the average prevalence rate of venous thromboembolism in the form of DVT and PE was reported to be 11.2% and 7.8%, respectively, whereas arterial thromboembolism was dominated by acute coronary syndrome (1.6%) and stroke (0.9%) [6]. Similar to other studies [4], the incidence of venous thromboembolism was increased by more than 2.5 times in COVID-19 patients who required treatment in the intensive care unit [6]. COVID-19 patients with complications of venous thromboembolism tend to have a higher risk of death (4.42 times higher for patients who are not hospitalized and 1.63 times higher for those who are hospitalized); meanwhile, COVID-19 patients with arterial thromboembolism undergoing treatment at a hospital have a 1.93 times higher risk of death, and, for those who are not hospitalized, the risk of death increases to 3.16 times higher than that of COVID-19 patients without arterial thromboembolic complications [5]. In addition to the short-term effects, the incidence of thrombosis in COVID-19 is also expected to have a significant impact on the quality of life of patients. In the non-COVID-19 population, for instance, more than 50% of patients with venous thromboembolism reported postthrombotic syndrome despite receiving adequate doses of anticoagulation therapy. These long-term complications have a major impact on quality of life and are closely associated with higher risks of depression, unemployment, social isolation, and healthcare-related costs [7,8,9]. 

Recently, data showed that the risk of venous thromboembolism (i.e., DVT and PE) also increases after a COVID-19 infection. For example, compared to the control period, the event rate ratio (i.e., the ratio of the event rate in the exposed group divided by the event rate in the unexposed comparison group) increased significantly up to 70 days after COVID-19 for DVT and 110 days for PE. Furthermore, the highest event rate ratio was found in patients with critical COVID-19 and was recorded to be higher in the first wave of the COVID-19 pandemic as compared to the second and third waves [10].

Taking into account all of the abovementioned data, here, we seek to discuss the clinical characteristics of COVID-19 infections, particularly with regard to the underlying pathophysiology; the pathogenesis of COVID-19-associated thrombosis at the molecular and cellular levels; the clinical manifestations of venous and arterial thromboembolism in COVID-19; and the potential benefits of several laboratory markers of thrombosis. Lastly, the preventive and therapeutic management of thromboembolism during and after COVID-19 will also be discussed. 

## 2. Clinical Characteristics of COVID-19 Infections

COVID-19 has a wide spectrum of clinical manifestations, ranging from asymptomatic to critical and life-threatening conditions. In general, adult patients infected with SARS-CoV-2 can be classified into several groups based on the severity of their experienced symptoms: (1) asymptomatic or presymptomatic infection occurs when a person obtains a positive result on the SARS-CoV-2 virus detection test, either using the antigen method or the nucleic acid amplification test (NAAT), but does not feel any specific symptoms of COVID-19; (2) mild illness occurs if a person has the typical symptoms and/or signs of COVID-19 (e.g., cough, fever, sore throat, weakness, headache, muscle aches, nausea, vomiting, diarrhea, hypo-/ageusia, and hypo-/anosmia) without being followed by more severe symptoms/signs, such as shortness of breath and abnormalities on a chest X-ray; (3) moderate disease occurs in patients who have lower respiratory tract abnormalities found during the physical examination or diagnostic studies but still have an oxygen saturation, as measured through pulse oximetry (SpO_2_), ≥ 94% at room temperature; (4) severe disease occurs in patients with an SpO_2_ < 94% at room temperature, a ratio of the arterial partial pressure of oxygen to the fraction of inspired oxygen (PaO_2_/FiO_2_) < 300 mmHg, a respiratory rate > 30 breaths per minute, or pulmonary infiltrates on a chest X-ray > 50%; and (5) COVID-19 falls into critical condition if the patient has experienced respiratory failure, septic shock, and/or multiorgan failure [11]. Furthermore, male gender, advanced age, obesity, a history of smoking, hypertension, diabetes mellitus, malignancy, cardiovascular disease (e.g., coronary artery disease), chronic liver disease, chronic obstructive pulmonary disease, and chronic kidney disease are independent risk factors for the occurrence of severe COVID-19, while acute respiratory distress syndrome, acute kidney disease, and shock are factors that hinder recovery after a COVID-19 infection [12,13,14].

The symptoms and signs that can occur in COVID-19 infections also vary from one individual to another and from one virus variant to another. A systematic literature review that analyzed 152 previous studies with a total sample of 41,409 individuals from 23 countries reported that fever, cough, shortness of breath, weakness, fatigue, and excessive sputum production were the symptoms most often complained of by people infected with COVID-19. Furthermore, nervous disorders, anorexia, muscle aches, sore throat, sneezing, runny nose, headaches, chest pains, and diarrhea are also common in people infected with COVID-19 [15]. Unlike the other variants of concern, the clinical signs and symptoms of Omicron variant infections are generally milder, with symptoms predominating in upper respiratory tract infections. Symptoms commonly reported in Omicron variant infections include fever, cough, sore throat, sneezing, and runny nose. Interestingly, reduced or absent taste and smell (ageusia and anosmia) are rarely seen in Omicron variant infections [16].

The variations in the symptoms and clinical signs could be related to the pathogenesis of the SARS-CoV-2 trajectory from entering the body to inducing clinical manifestations. The SARS-CoV-2 virus is transmitted via respiratory droplets and aerosols with an incubation period of 4–5 days. Generally, the cells that are first attacked by SARS-CoV-2 in humans are the multiciliated cells in the nasopharynx and trachea or the sustentacular cells in the nasal mucosa. The virus binds to the angiotensin-converting enzyme type 2 (ACE2) receptor, and, with the help of transmembrane serine protease 2 (TMPRSS-2), there is an activation of the S2 subunit and fusion between the viral and host membranes [17]. After entering the cell, the viral genetic material then initiates the production of viral proteins, including the replicase protein, which plays a role in the formation of the virus replication “factory” in the host’s body. The vesicles in which the transcription of the viral genetic material (i.e., double-stranded RNA (dsRNA)) takes place are protected by a double membrane that allows them to escape detection by cytoplasmic pattern recognition receptors (PRRs). If the dsRNA can be recognized by the PRR (e.g., by melanoma differentiation-associated protein 5, or MDA5), a signal cascade occurs that initiates the production of interferon I and III [18]. Apart from the initiation of the PRR, interferons and chemokines can also be produced as a result of the local responses of the epithelial cells surrounding the host cells and local immune cells, such as neutrophils and macrophages. Then, the interferons produce genes that have cellular antiviral capabilities, which are intended to eliminate SARS-CoV-2 from the cell. At the same time, the released cytokines activate B- and T-lymphocyte cell responses to facilitate virus elimination [19]. When the virus is in the upper respiratory tract, the clinical signs and symptoms of an upper respiratory tract infection can be found, which are typically observed in the early phase of COVID-19 infections or in patients with mild symptoms.

If the innate and adaptive immune system is unable to eliminate the virus from the upper respiratory tract, the virus spreads to the lower respiratory tract through the inhalation process or spreads slowly from top to bottom through the bronchi and its branches. If the virus reaches the lower respiratory tract, it can cause dyspnea, damage to the alveoli, and impaired gas exchange caused by inflammation induced by virus invasion into the cells [20]. Within the alveoli, the SARS-CoV-2 virus infects the type 2 alveolar/pneumocytic cells (AT2s), which physiologically produce surfactants which are important for reducing the surface tension of the alveoli in respiration and which are the AT1 progenitor cells in adult lungs. Therefore, virus invasion into the AT2s can also cause impaired gas exchange, which is predominantly modulated by the AT1s [19,21]. Severe illness generally occurs after one week from the onset of symptoms, but the progression of COVID-19 is greatly influenced by intrinsic and extrinsic cofactors, such as comorbidities, age, immune status, medications, and others.

## 3. Coagulopathy in COVID-19 Infections

Coagulopathy is one of the major clinical manifestations of COVID-19. Several mechanisms are involved in the pathogenesis of this complication, including endothelial injury, hyperinflammatory response and cytokine storm, complement activation, mononuclear phagocytes, neutrophil extracellular trap (NET) formation, and tissue hypoxia [22,23,24].

### 3.1. Endothelial Injury and Dysfunction

**Figure 1:**
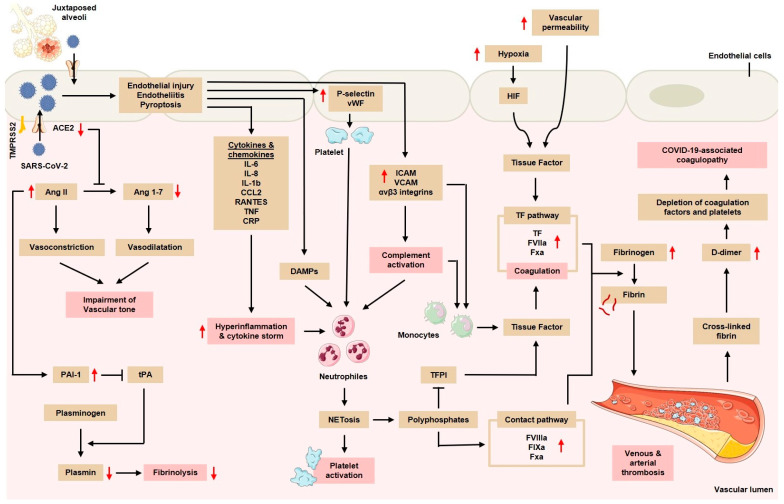
Pathogenesis of COVID-19-associated thrombosis. SARS-CoV-2 invades vascular endothelium, causing endothelial injury, endotheliitis, and NLRP3-mediated pyroptosis. These processes release proinflammatory cytokines and chemokines, inducing hyperinflammation in the vascular lumen. The activated neutrophils then stimulate the formation of NETs, allowing platelet activation and producing polyphosphates that activate contact pathway. Together with the activation of TF pathway, they facilitate the conversion of fibrinogen into fibrins, which, in turn, increase the risk of COVID-19-induced coagulopathy, including thrombosis. (ACE2 = angiotensin-converting enzyme type 2; Ang II = angiotensin II; Ang 1-7 = angiotensin 1-7; CCL2 = C-C chemokine ligand 2; CRP = C-reactive protein; DAMP = damage-associated molecular pattern; HIF = hypoxia-induced transcription factor; ICAM = intercellular adhesion molecule 1; IL = interleukin; NET = neutrophil extracellular trap; PAI-1 = plasminogen activator inhibitor-1; RANTES = regulated on activation, normal T cell expressed and secreted; TFPI = tissue factor pathway inhibitor; tPA = tissue plasminogen activator; VCAM = vascular cell adhesion molecule 1; and vWF = von Willebrand factor).

SARS-CoV-2 can directly invade the endothelial cells in the lung microvasculature. This is tightly associated with the viral tropism of SARS-CoV-2 in the cells that express abundant ACE2, for example, in the AT2 cells. Because the location of the AT2s is adjacent to the pulmonary vascular bed, the virus can migrate and attack the juxtaposed vascular endothelial cells. As a result, endothelial cell hyperplasia, resulting from the blockage of small blood vessels by inflammatory cells and by a thrombus in large blood vessels, is often found. Additionally, several studies proposed that SARS-CoV-2 could directly infect vascular endothelial cells and cause cell damage and apoptosis, thereby reducing the antithrombotic activity of the normal endothelium [25,26,27,28,29]. Such a reduction in antithrombotic activity is marked by an increase in von Willebrand factor (vWF), fibrinogen, and factor VIII in COVID-19 patients [22,23]. However, several other studies later showed that endothelial cells expressed very low or negligible *ACE2* as compared to the epithelial cells from the respiratory, gastrointestinal, or skin tissues, raising a doubt in the data demonstrating the direct invasion of SARS-CoV-2 into the endothelium via the ACE2 receptor [30,31,32]. A study attempting to disentangle this controversy showed thereafter that the endothelial cells of the human pulmonary artery possessed ACE2 proteins capable of interacting with the spike protein of SARS-CoV-2. However, the intact glycocalyx of the endothelium inhibited such an interaction, thereby preventing contact between the virus and ACE2 receptor [28]. Regardless of the controversy, patients infected with SARS-CoV-2 were reported to have increased levels of IL-6, IL-1β, interferon (IFN)-γ, monocyte chemoattractant protein 1 (MCP-1), macrophage inflammatory protein (MIP), and IP10 (CXCL10). These proinflammatory cytokines can interfere with endothelial function and integrity, leading to a vWF release; the upregulation of adhesion molecules, such as intercellular adhesion molecule 1 (ICAM-1), αvβ3 integrins, and P- and E-selectins; and the production of endothelial cytokines and chemokines (Figure 1) [24,33]. In addition, SARS-CoV-2 can also infect pericytic cells, which play a role in vascular homeostasis and the regulation of the inflammatory processes [34]. As a result, the inflammation of the endothelium (i.e., endotheliitis) can occur. Several reports of patients who died from COVID-19 showed an accumulation of inflammatory cells and viral inclusions, which were detected through histology and electron microscopy. In addition, in autopsy and surgical tissue specimens, diffuse lymphocytic endotheliitis and apoptotic bodies were present. The tropism of SARS-CoV-2 in the cells with high numbers of ACE2 receptors together with the anatomical position of the AT2s, which are close to the pulmonary vascular network, can result in a severe inflammatory reaction, which can lead to widespread pulmonary hypercoagulability [35]. Previous autopsy studies showed that, in patients who died from COVID-19, there was an endothelial injury accompanied by thrombosis and extensive microangiopathy [36].

Increased levels of vWF, which is an endothelial adhesion protein, were reported in both COVID-19 patients who were treated in intensive care units and in patients who did not fall into critical condition, and this increase was associated with the decreased activity of a disintegrin and a metalloproteinase with a thrombospondin type 1 motif, member 13 (ADAMTS13), a metalloproteinase that regulates the size of the vWF multimer [37,38]. The disturbance of the endothelial cells together with the release of large vWF multimers and the relatively inadequate cleavage of vWF due to a decreased ADAMTS13 can lead to increased interactions between the blood vessel walls and platelets, which then lead to thrombotic microangiopathy [25]. High numbers of circulating endothelial cells have been reported in patients with COVID-19, especially in patients requiring intensive care [39], and the presence of circulating endothelial cells is positively correlated with platelet and lymphocyte counts and with soluble vascular cell adhesion molecule 1 (sVCAM1), which is a classic endothelial marker. Another report from Italy has also confirmed that COVID-19 patients have an increased number of circulating endothelial cells, with increased plasma levels of soluble ICAM1 (sICAM1) and sVCAM1, indicating the occurrence of endothelial dysfunction in COVID-19 infections [25,40].

### 3.2. Hyperinflammatory Response and Cytokine Storm

In COVID-19 patients, it is common to observe elevated serum levels of proinflammatory cytokines and chemokines (Figure 1), especially in severe and fatal COVID-19 cases. The cytokine profiles in patients with severe COVID-19 infection showed an increased production of IL-6; IL-7; tumor necrosis factor (TNF); and inflammatory chemokines, such as the C-C chemokine ligand 2 (CCL2) motif, C-C chemokine ligand 3 (CCL3) motif, and soluble IL-2 receptors, which are similar to those found in cytokine release syndrome (CRS), e.g., macrophage activation syndrome. Excessive cytokine release can cause thrombosis through various mechanisms, including the activation of monocytes, neutrophils, and the endothelium, all of which facilitate a prothrombotic state [22]. IL-1α, which is widely expressed by activated platelets, endothelial cells, and circulating monocytes during proinflammatory conditions, serves as a link between the coagulation cascade and the inflammatory response. IL-1α plays a role in thrombosis by causing an increase in the clot lysis time, increasing platelet activity, and activating the endothelial cells. The release of IL-1α by the epithelial cells as alarmin is followed by the sensing of the inflammatory myeloid cells and the activation of the inflammasome, resulting in the amplification of the inflammatory cascade [41]. Simultaneously, the expression of IL-1α by the endothelial cells leads to the recruitment of granulocytes and thrombosis [42]. 

### 3.3. The Activation of Mononuclear Phagocytes, Complements, and NETs

After the recognition of the viral components via the PRR, monocytes and the microvesicles produced by monocytes present activated tissue factor (TF) on their surface and release it at the localization site of the pathogen, activating the extrinsic coagulation pathway [43]. In addition, pathogens also stimulate the activation of NOD-, LRR-, and pyrin-domain-containing protein 3 (NLRP3) in monocytes and/or macrophages, leading to the release of proinflammatory cytokines, such as interleukin-1β (IL-1β) and IL-18 [44,45]. Neutrophils are then recruited and contribute to this process through the release of NETs, which directly activate factor XII (Hageman factor). NETs also bind to vWF and help recruit platelets. Histones, particularly H3 and H4, trigger platelet activation. In addition, the neutrophil elastase and myeloperoxidase (MPO) in NETs bypass and inactivate natural anticoagulants (i.e., tissue factor pathway inhibitors (TFPIs) and thrombomodulin (TM)). Then, NETs externalize and bind to TF, facilitating the activation of the extrinsic coagulation pathway. Platelets support the immunothrombotic process by activating the contact-dependent coagulation pathways via a polyphosphate release and, together with the endothelial cells, can promote fibrin formation. Platelets can also be activated by the complement system (C3a and C5a). Activated platelets release large amounts of proinflammatory cytokines within the platelet extracellular vesicle (PEV) [46,47]. Through this mechanism, pathogens such as SARS-CoV-2 are trapped in the fibrin-based NETs and are then killed. The immunothrombotic process allows for the elimination of pathogens to be confined to the intravascular compartment, thus limiting injury to organs [25].

### 3.4. Tissue Hypoxia

Hypoxia is one of the determinants of thrombosis in COVID-19. Previous studies have shown that the vascular response to hypoxia is controlled by hypoxia-induced transcription factors (HIFs; Figure 1). HIFs, including HIF-1α, are known to trigger thrombosis via plasminogen activator inhibitor (PAI)-1 and TF [48]. In addition, HIF-2 has a prothrombotic effect by inhibiting the action of TF inhibitors. HIF-1α is expressed in the alveolar epithelial cells and has been shown to trigger cellular inflammation through the release of proinflammatory/prothrombotic cytokines, such as TNFα and IL-6. In addition, hypoxia is thought to have a direct prothrombotic effect on the endothelium, such as a suppressive effect on thrombomodulin and decreased fibrinolytic function [23]. Vascular hypoxia induces HIF activation and increases endothelial TF expression along with the downregulation of natural anticoagulants, Protein S, and TFPI [24].

## 4. Clinical Manifestations of Thrombosis in COVID-19

### 4.1. Venous Thromboembolism

#### 4.1.1. Deep-Vein Thrombosis

Deep-vein thrombosis (DVT) is an obstructive disease that results from the inhibition of the venous reflux mechanism. DVT usually involves the venous system of the lower extremities, with clot formation originating in the deep veins of the femur and spreading proximally [49]. In patients with a COVID-19 infection, the incidence of DVT was recorded at 14.8% [50]. Other studies have also reported similar event magnitudes, especially in patients with elevated D-dimer. Elevated D-dimer levels, a history of previous or active cancer, and the need for a high-flow nasal cannula (HFNC) or noninvasive ventilation were significantly associated with the development of DVT in patients with a COVID-19 infection. In addition, the length of stay in the hospital due to COVID-19 is also directly proportional to the risk of DVT. For example, the prevalence of DVT on days 7, 14, and 21 was 16%, 33%, and 42%, respectively. This is very likely due to the many complications and severity of COVID-19 experienced by patients with prolonged hospitalization. Indeed, an increased incidence of DVT was found in critically ill COVID-19 patients with high D-dimer, particularly in patients requiring intensive care, intubation, and mechanical ventilation [23].

Interestingly, the reported risk of developing DVT remains high after COVID-19 infection. Data show that the incidence rate ratio increases up to 70 days after COVID-19, while the risk ratio in the first 30 days after COVID-19 reaches 4.98 [10]. Furthermore, another study conducted in England analyzed the hazard ratio (HR) of DVT in COVID-19 patients compared to non-COVID-19 patients. As a result, in the first week of the diagnosis of COVID-19, the HR of DVT reached 10.8, which means that the risk of COVID-19 patients experiencing DVT in the first week after diagnosis is 10.8 times higher than that of non-COVID-19 patients. Interestingly, even though there was a significant decrease in the following weeks, the HR of DVT at week 49 after the diagnosis of COVID-19 was still 1.99; on the other hand, there was still a 2-fold increased risk of DVT at week 49 after the diagnosis of COVID-19, and there was no difference between the HR of DVT at week 49 in patients who had previously been hospitalized due to COVID-19 compared to that in patients who had not [51].

#### 4.1.2. Pulmonary Embolism

Pulmonary embolism (PE) occurs when there is an obstruction to the blood flow in the pulmonary artery or its branches caused by a thrombus originating elsewhere. PE often results from the release of a thrombus formed in DVT into the general circulation, which eventually occludes pulmonary circulation. Therefore, the risk factors for PE are identical to those of DVT, namely, the presence of disorders in Virchow’s triad (i.e., intravascular vessel wall damage/vascular endothelial damage, flow stasis, and the presence of a hypercoagulable state). PE causes impaired gas exchange due to a pulmonary vascular obstruction, leading to a mismatch in the ventilation/perfusion ratio because alveolar ventilation remains the same while pulmonary capillary blood flow decreases, effectively causing dead space ventilation and hypoxemia [52]. Apart from DVT, PE is also often found in patients infected with COVID-19, especially in patients who are hospitalized due to severe or critical COVID-19 [23]. A study evaluating 413 patients with COVID-19 reported that PE occurred in 102 of 413 (25%) hospitalized patients, with a higher percentage of patients requiring intensive care (29%) than those who did not (24%). The study stated that male sex; smoking; and increased levels of D-dimer, lactate dehydrogenase (LDH), ferritin, and IL-6 are risk factors for PE in COVID-19. Furthermore, D-dimer levels > 1600 ng/mL can help predict PE with a sensitivity of 100% and a specificity of 62% [53]. A multicenter retrospective study involving 1240 COVID-19 patients reported an incidence of PE of 8.3% and, interestingly, the administration of prophylactic doses of anticoagulants or anticoagulant therapy was a protective factor for COVID-19 patients (odds ratio of 0.83–0.87), whereas male sex, increased C-reactive protein (CRP), and the days from the onset of symptoms to hospitalization were independent predictors of PE [54].

Similar to DVT, data show that the risk of PE after COVID-19 infection is still high up to 110 days after COVID-19. In particular, the incidence rate ratio was higher for male patients compared to female patients during the first 3 months after COVID-19 and was highest in the 50–70 year age group [10]. Another study conducted in the UK analyzed the HR of PE in COVID-19 patients compared to non-COVID-19 patients. As a result, in the first week after the diagnosis of COVID-19, the risk of PE increased 33 times and gradually decreased to 1.61 times in the 49th week. Consistently, even after adjusting for age, sex, and region, the adjusted HR (aHR) of PE at week 49 still showed an increase in the COVID-19 group (aHR of 2.22 and 95% CI of 1.69–2.92), which was dominated by a subgroup of patients who had not previously been hospitalized due to COVID-19 [51].

### 4.2. Arterial Thromboembolism

#### 4.2.1. Acute Myocardial Infarction

A study in Sweden involving 88,742 COVID-19 patients reported that COVID-19 was an important risk factor for acute myocardial infarction (AMI) during the pandemic, with an incidence rate ratio of 2.89 in the first week of COVID-19 infection, which decreased to 1.60 in the third and fourth weeks of COVID-19 infection [10]. Another study also reported that the risk of AMI approximately doubled within 7 days after the diagnosis of COVID-19. Various studies have shown worse outcomes in patients with COVID-19 and MI. This is mainly related to the pathogenesis described above, with the direct effect of SARS-CoV-2 on the endothelial cells and the increased tendency towards vascular thrombosis in COVID-19 [55]. For example, the mortality rate for STEMI patients infected with COVID-19 reached 77%, while, in patients without COVID-19, the mortality rate “only” reached 44% [56]. Similar to the complications of venous thromboembolism in COVID-19, the incidence of AMI has also been reported to be high after a COVID-19 infection. In the first week after the diagnosis of COVID-19, the HR of AMI in COVID-19 patients reached 17.2 and decreased to 1.21 at week 49. This data illustrates that, after almost 1 year after the diagnosis of COVID-19, there is still an increased risk (increased by 21%) of AMI in patients who have been infected with COVID-19 when compared to patients who have not been infected with COVID-19. This increased risk was especially experienced by patients with a history of hospitalization due to COVID-19 [51]. 

#### 4.2.2. Ischemic Stroke

Ischemic stroke is often associated with COVID-19 and, in previous studies, it was stated that 4.6% of COVID-19 patients experienced acute ischemic stroke. The clinical risk factors associated with ischemic stroke in patients infected with COVID-19 are age, disease severity, cardiovascular risk factors, increased CRP, and increased D-dimer levels [57]. A meta-analysis that included 61 articles with a total sample of 108,571 COVID-19 patients reported that there was an incidence of stroke of 1.4%, which was dominated by ischemic stroke (87.4%). The associated risk factors included old age, hypertension, diabetes mellitus, coronary heart disease, and severe COVID-19 [58]. Consistent with the other thromboembolic complications of COVID-19, the incidence of ischemic stroke has also been reported to be high after a COVID-19 infection, with an HR of 23 at week 1, which decreased to 1.62 at week 49. The persistence of the risk of ischemic stroke after a COVID-19 infection was seen both in patients who had previously been hospitalized due to COVID-19 and in those who had not [51].

## 5. Diagnostic Markers of Thrombosis in COVID-19 Patients

### 5.1. Platelet Counts and Immature Platelet Fraction

Platelets are the second most abundant circulating cells, with > 1 trillion cells circulating in the vessels at one time. In addition to their role in hemostasis, platelets also have an important role in the regulation of the immune system. Activated platelets release a number of chemokines from their dense and alpha granules to facilitate the recruitment of leukocytes to the sites of vascular injury/inflammation, including CXCL1; platelet factor 4/CXCL4 (PF-4); CXCL5; NAP-2 (CXCL7); CCL3; regulated on activation, normal T cell expressed and secreted (RANTES); CCL5; and CCL7. The release of chemokines by platelets facilitates leukocyte recruitment and adhesion to thrombi, also serving to modulate the functional response of leukocytes. In addition, platelets also express IL-1β mRNA, which can be translated and secreted as mature IL-1β by activated platelets. Activated platelets also express P-selectin, which allows leukocyte tethering via P-selectin glycoprotein ligand 1 (PSGL-1), which is expressed by platelets. Platelets also have the ability to migrate, eat, and bind bacteria; then, they present them to neutrophils for phagocytosis and trigger an NET release, which can be induced by P-selectin/PSGL-1 as well as by the interaction of the GPIIb/IIIa (αIIbβ3, CD41/CD61) of platelets with SLC44A2 (CTL-2) on neutrophils. In addition, activated platelets can release lipid-rich microvesicles that can transport various cytokines and chemokines, such as IL-1β, CXCL4, CXCL7, and CCL5; growth factors; microRNAs; and mitochondria [24]. 

Mild thrombocytopenia has been reported as a common finding in severe COVID-19. A meta-analysis showed that thrombocytopenia caused a 5-fold increase in the risk of disease severity as well as increased the risk of death in patients infected with COVID-19 [59]. A consensus issued by the International COVID-19 Thrombosis Biomarkers Colloquium states that platelet counts can be used to determine the prognosis of COVID-19. A low platelet count may reflect a poorer prognosis. However, there are no data reporting the benefits of calculating platelet counts for predicting thrombotic events in COVID-19 [60]. The International Society of Thrombosis and Hemostasis in its 2020 interim report stated that the trend towards a decreased platelet count over time in patients with COVID-19 might indicate a worsening thrombotic status [61]. Conversely, improvement in thrombocytopenia in COVID-19 patients could also reflect clinical improvement in the disease [62].

The number or fraction of immature platelets (IPF, or reticulated platelets) can also be used to determine the risk of needing intensive care in patients infected with COVID-19 [60]. The number of reticulated platelets is also positively related to cardiovascular risk and death. In healthy adults with normal platelet counts, the IPF ranges from 3.3 to 8.6%. There is a trend towards a significant increase in the IPF in COVID-19 patients. Data from four COVID-19 patients showed an absolute IPF of 7.5 × 10^9^/L or higher and a relative IPF of ≥ 8% with a platelet count of up to 251 × 10^9^/L. Of note, in non-COVID-19 patients, a relative IPF of ≥ 8% is usually found in patients with a platelet count of less than 70 × 10^9^/L. These findings suggest that, in COVID-19, there is an increase in the production of immature platelets as a response of megakaryocytes to the increased use of platelets [61].

### 5.2. D-Dimer

Dimerized plasmin fragment D (D-dimer) is a fibrin degradation product (FDP) which is formed in the process of hemostasis. Throughout blood clotting activation, fibrinogen molecules are continuously converted into fibrin monomers that passively aggregate via an enzymatic process catalyzed by transglutaminase-activated factor XIII (FXIIIa) until covalent bonds are formed between adjacent fibrin monomers to create a stable fibrin network in which platelets and other blood cells are trapped. More specifically, FXIIIa catalyzes the formation of an isopeptide bond between the γ-carboxyamine group of the glutamines and the ε-amino group of the lysine located on the two fibrinogen D domains present on the two adjacent fibrin monomers. After hemostasis has completed its task of restoring the integrity of the blood vessels, the process of fibrinolysis is activated to lyse the remaining blood clots that have formed. This process is characterized by the degradation of stable fibrin by the enzyme plasmin, which can be activated from its precursor plasminogen by a number of endogenous (e.g., urokinase and tissue plasminogen activator (tPA)) or exogenous (e.g., recombinant tPA) enzymes. The degradation of stable fibrin produces a heterogeneous mixture of so-called FDPs, which generally vary in size and composition, one of which is D-dimer. Because the formation of D-dimer only occurs through the breakdown of stable fibrin, D-dimer is considered to be a very specific biomarker of fibrin degradation [63]. A normal amount of cross-linked FDPs is produced under physiological conditions, and the amount increases with age. This is why D-dimer can also be detected in healthy people and why the aging process increases the blood levels of D-dimer. In general, a normal D-dimer concentration is about 500 μg/L, or 0.5 mg/dL fibrinogen equivalent unit (FEU; 1 μg/L FEU = 0.5 μg/L D-dimer unit [DDU]) [63].

In COVID-19, there is often an increase in the concentration of serum D-dimer, indicating the occurrence of thrombosis in the pulmonary vascular tissue and fibrinolysis. This significant increase in D-dimer in COVID-19 reflects not only coagulation activity due to viremia and cytokine storm but also that due to superinfection and organ dysfunction. A cut-off value of D-dimer > 1000 μg/L can help stratify COVID-19 patients with a poor outcome risk. D-dimer levels > 1.0 µg/mL on admission are associated with an increased risk of mortality. An increase in D-dimer levels over time can also indicate a progressive degree of severity of a COVID-19 infection and can be used as a marker for the need for more aggressive management of COVID-19 [62,64]. Besides its benefits as a marker of disease severity, D-dimer can also be used to predict the occurrence of the complications of venous and arterial thromboembolism in COVID-19 infections [23,65]. A study conducted on 158 patients infected with COVID-19 (52 patients with DVT and 106 without DVT) reported higher acute-phase D-dimer levels in the DVT group (median of 13,602 ng/mL) compared to the non-DVT group (median of 2880 ng/mL). Furthermore, a D-dimer cut-off value of 6494 ng/mL was claimed to be used to separate patients who had DVT from those who did not (sensitivity of 80.8% and specificity of 68.9%) [66]. Similar to DVT, D-dimer concentrations were also higher in patients with PE than those without PE (9.1 µg/mL vs. 2.3 µg/mL); additionally, with a cut-off value of 0.5 µg/mL or higher, the sensitivity of PE prediction reached 98.2%, but the specificity only reached 5.7% [67]. Similar results were obtained in different studies with 100% sensitivity, 9.3% specificity, and 21% accuracy at a cut-off value of 0.5 μg/mL, indicating that the use of a D-dimer assay at the designated cut-off value to rule out PE among hospitalized COVID-19 patients was less accurate [68].

The D-dimer value not only has clinical significance in the acute phase of infection, but emerging evidence also seems to support the long-term prognostic role of D-dimer, as a large number of patients, especially those with severe COVID-19, may still have a high D-dimer value long after remission and hospital discharge. A total of 222 COVID-19 patients who were followed for 12 months showed a relationship between high D-dimer levels and an increased risk of death (by 60%) in the first year [63,69]. It was previously reported that a D-dimer level > 3 μg/mL together with an inpatient CRP level > 10 mg/dL and a previous history of venous thromboembolism increase the risk for posthospital venous thromboembolism and that the administration of anticoagulant therapy on discharge can reduce the risk of venous thromboembolism after hospitalization [70]. Taking into account the previously published data, a consensus from the International COVID-19 Thrombosis Biomarkers Colloquium states that D-dimer can be used to determine the diagnosis and prognosis of thromboembolism related to COVID-19 but is not optimal for evaluating the effectiveness of anticoagulation therapy because large-sample prospective studies consistently reported the benefit of anticoagulation in thrombosis independent of the D-dimer levels. However, the benefits of anticoagulation therapy are indeed greater in patients with high D-dimer levels [60].

### 5.3. Von Willebrand Factor

Activated or damaged endothelial cells release Weibel–Palade bodies, which contain ultralarge-molecular-weight vWF multimers that can spontaneously bind to platelets and cause microthrombosis if not cleaved by ADAMTS13. It is known that COVID-19 patients experienced significantly increased levels and function of vWF together with increased FVIII clotting activity, while ADAMTS13 activity slightly decreased [62]. Moreover, the markers of activation of the endothelial cells and platelets (i.e., vWF antigen and soluble P-selectin) were significantly increased in COVID-19 patients treated in the intensive care unit compared to those who did not require intensive care [71]. A case report reported increased plasma antigen and vWF activity as well as an increased FVIII activity level on day 21 of hospitalization in a critically ill COVID-19 patient, indicating endothelial cell injury. This report also noted an improvement in D-dimer levels after the onset of heparin therapy [72]. In addition, the levels of soluble vWF antigen and thrombomodulin were positively associated with the mortality rate of COVID-19 patients [71]. Therefore, based on the results of previous studies, a consensus by the International COVID-19 Thrombosis Biomarkers Colloquium concluded that vWF and the ratio of vWF antigen to ADAMTS13 have benefits for determining the prognosis of thromboembolism in COVID-19 but are less optimal for predicting the risk of thrombosis [60].

### 5.4. Viscoelastic Test and Thromboelastography

Thromboelastography (TEG) is a method to dynamically measure the viscoelastic properties of coagulation that is widely used in critical care, emergency care, and surgery, particularly to guide plasma and platelet transfusions in bleeding patients. Several studies have been able to detect hypercoagulability in COVID-19 patients using TEG or rotational thromboelastometry (ROTEM). For example, out of 21 COVID-19 patients in the intensive care unit, 90% of patients were hypercoagulable based on a TEG analysis [73]. Meanwhile, another study on critically ill COVID-19 patients who had received thromboprophylaxis reported a hypercoagulable incidence of 30.8% and an increase in the maximum amplitude (MA) of TEG in 75% of patients [74]. A lower MA is associated with an increased risk of venous thromboembolism in COVID-19. Each 1 mm increase in baseline and peak MA was associated with a reduced risk of venous thromboembolism (reduced by 8% and 14%, respectively). Lower initial platelet counts and fibrinogen levels are also associated with an increased risk of venous thromboembolism, while platelet counts and fibrinogen levels are positively related to MA [75]. A consensus of the International COVID-19 Thrombosis Biomarkers Colloquium concluded that the viscoelastic test as a marker of coagulation and fibrinolysis may be useful in determining the prognosis of COVID-19-associated thrombosis. Preliminary data support the use of TEG to personalize antiplatelet or antithrombotic therapy to improve outcomes, but more data is needed before it can be applied in routine clinical practice [60].

### 5.5. Antiphospholipid Antibody

COVID-19 infections and antiphospholipid syndrome (APS) share numerous similarities pathophysiologically. Both can cause multiorgan disorders accompanied by increased levels of LDH and D-dimer and a decreased platelet count (thrombocytopenia). Both entities may also develop thrombotic microangiopathy through endothelial injury, complement activation, and NET release (NETosis). Early studies on COVID-19 patients receiving DVT prophylaxis who were hospitalized for thrombosis noted a prolonged activated partial thromboplastin time (aPTT) and positive antiphospholipid antibody (APL), especially in critically ill patients. There are three APS classification criteria tests for detecting APL: the lupus anticoagulant (LAC) test, IgG/M aβ2GPI test, and IgG/M anticardiolipin (ACL) test. The LAC test is positive in 5–90% of patients infected with COVID-19, while a positive test for IgM anticardiolipin is present in 3–23%; additionally, a positive test for IgG is present in 5–13% of COVID-19 patients who fall in critical condition, while IgM aβ2GPI is present in 2–16% and IgG in 3–18% of patients. However, patients with a clinically significant APL profile (i.e., high aCL/aβ2GPI titers or triple-positive APL (positive LAC, ACL, and aβ2GPI)) are rare [76]. To date, the contribution of APL to COVID-19-associated coagulopathy remains unclear. Most studies reported the presence of one or more APL markers, but it has not been confirmed whether there is a correlation with the thrombotic process or coagulopathy. Further research is still needed to determine the role of APL in thromboembolism related to COVID-19 [23].

## 6. Prevention and Management of Thrombosis in COVID-19

### 6.1. Thrombosis Management

#### 6.1.1. Anticoagulation

**Figure 2:**
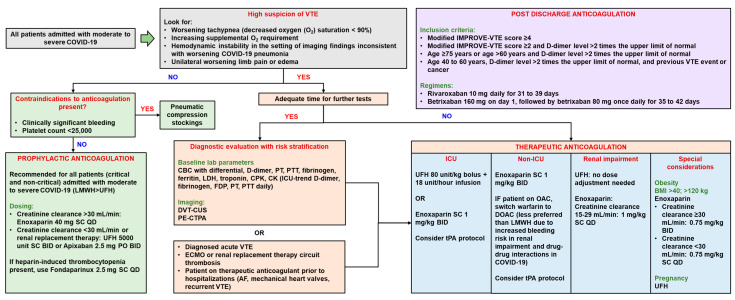
Proposed workflow of the management of COVID-19-associated thromboembolism by Hajra et al. [78]. (AF = atrial fibrillation, BID = twice a day, BMI = body mass index, CBC = complete blood count, CK = creatinine kinase, CPK = creatinine phosphokinase, CTPA = computed tomography pulmonary angiography, CUS = compression ultrasonography, DOAC = direct oral anticoagulant, DVT = deep-vein thrombosis, ECMO = extracorporeal membrane oxygenation, ICU = intensive care unit, IMPROVE = International Medical Prevention Registry on Venous Thromboembolism, LDH = lactate dehydrogenase, LMWH = low-molecular-weight heparin, OAC = oral anticoagulation, PE = pulmonary embolism, PT = prothrombin time, PTT = partial thromboplastin time, QD = once a day, SC = subcutaneous, tPA = tissue plasminogen activator, UFH = unfractionated heparin, and VTE = venous thromboembolism).

Therapeutic doses of anticoagulation therapy (Figure 2) are beneficial for patients who meet the criteria for sepsis-induced coagulopathy (SIC [77]) or who have high levels of D-dimer [24]. A retrospective study involving 449 severe COVID-19 patients reported a reduction in mortality at day 28 in patients with an SIC score ≥ 4 and D-dimer > 3.0 μg/mL who were treated with heparin compared to those who were not. In addition, therapeutic doses of anticoagulants are also recommended for COVID-19 patients who have experienced thromboembolic incident(s) or who have a high suspicion of thromboembolic disease. COVID-19 patients who require extracorporeal membrane oxygenation or ongoing renal replacement therapy or who have an extracorporeal catheter or filter thrombosis should be given therapeutic anticoagulants according to the standard institutional protocols [78,79]. In agreement, the National Institute of Health (NIH) released a treatment guideline for antithrombotic therapy in patients with COVID-19 [79] and also recommended therapeutic anticoagulation in COVID-19 patients with a high suspicion of thromboembolic disease if confirmatory diagnostics cannot be performed. Heparin is known not only to inhibit blood clotting through the inhibition of factor Xa or thrombin through its binding to antithrombin but is also reported to have pleiotropic effects, such as anti-inflammatory effects, due to the formation of bonds with danger-associated molecular patterns (DAMPs), such as high mobility group box 1 (HMGB-1), and proinflammatory cytokines as well as SARS-CoV-2 inhibitory effects on it binding to the host cells in in vitro studies [24].

Apart from heparin-based anticoagulants, DOAC can still be used in COVID-19 patients as indicated. However, DOAC can interact with anti-IL-6 (tocilizumab) and antivirals (lopinavir, ritonavir, or darunavir), which are often used in the treatment of COVID-19. In addition, impaired kidney function, which is often found in critically ill COVID-19 patients, reduces DOAC excretion, increasing the risk of bleeding [78].

#### 6.1.2. Tissue Plasminogen Activator (tPA)

Tissue plasminogen activator (tPA) converts the inactive plasminogen enzyme into plasmin, which causes the breakdown of cross-linked fibrin (fibrinolysis). A case series reported the effects of tPA in COVID-19 patients with ARDS and respiratory failure and reported an initial increase in the ratio of inspired partial pressure oxygen to fractional oxygen (PaO_2_/FiO_2_). However, the effect was only temporary [78,80,81]. In addition, the potential benefits of tPA are overshadowed by the significant risk of major bleeding. This argument provides a basis for locally administered (nebulized) tPA therapy to increase local concentrations and reduce systemic adverse effects. Interestingly, the data suggested that, in severe ARDS, nebulized fibrinolytic therapy was associated with improvements in the oxygenation and ventilation parameters [24,82]. In general, further research is still needed to evaluate the effects of tPA in COVID-19 patients, particularly in patients with vascular thromboembolic complications.

#### 6.1.3. Other Potential Therapies

The many mediators, cytokines, chemokines, and molecules involved in the pathogenesis of thrombosis in COVID-19 provide potential room for new, more effective therapies to treat the complications of thromboembolism in patients infected with COVID-19. For example, several therapies with antiplatelet effects, including dipyridamole and nafamostat, are currently being evaluated for their potential in reducing the severity of COVID-19. Antiplatelet dipyridamole was reported to be able to suppress the replication of SARS-CoV-2 in vitro, with some preliminary data suggesting that the use of dipyridamole as an adjunctive therapy yielded clinical improvements in patients with severe COVID-19. In addition, the role of nafamostat, a serine protease inhibitor that has antiplatelet effects and is currently being used for the treatment of disseminated intravascular coagulation (DIC) and pancreatitis, is also being evaluated. Nafamostat is known to inhibit TMPRSS-2, and, in preclinical studies, serine protease inhibitors were able to inhibit SARS-CoV-2 infections in the lung cells [24,83]. Unfortunately, a randomized controlled trial (RCT) of 21 COVID-19 patients who were hospitalized and who received intravenous nafamostat mesylate therapy did not record any anti-inflammatory, anticoagulation, or antiviral activity as previously observed in the reported preclinical studies [84]. Similarly, a phase II clinical trial on 104 hospitalized COVID-19 patients also documented no significant difference in terms of time to clinical improvement between the nafamostat and standard therapy groups. However, in a subgroup analysis of high-risk COVID-19 patients (National Early Warning Score (NEWS) baseline of ≥ 7) requiring oxygen supplementation, nafamostat therapy significantly increased their rate of recovery compared to the standard therapy [85].

### 6.2. Thrombosis Prevention

In 2022, the International Society on Thrombosis and Hemostasis (ISTH) issued guidelines for antithrombotic therapy for COVID-19, one of which recommended the use of prophylactic doses of anticoagulants in COVID-19 patients who are at high risk of thromboembolic events. Those included 5000 units of unfractionated heparin (UFH) injected subcutaneously two to three times daily, 40 mg of low-molecular-weight heparin (LMWH) enoxaparin injected subcutaneously once daily, 5000 IU of LMWH dalteparin injected subcutaneously once daily, 4500 IU of LMWH tinzaparin injected subcutaneously once daily, and 3500 IU of LMWH bemiparin injected subcutaneously once daily. In patients who are hospitalized with noncritical COVID-19, a prophylaxis with low-dose LMWH or UFH can be given to reduce the risk of thromboembolism and death. This recommendation was based on several previous observational studies which reported that, in hospitalized COVID-19 patients, low-dose prophylactic LMWH/UFH compared to no LMWH/UFH reduced mortality by 24% to 82%, and an observational study showed a reduced absolute risk (ARR) of thromboembolic events or death (reduced by 11.4%) following the use of prophylactic heparin compared to no anticoagulants. The overall risk of venous thromboembolism in noncritically ill patients who were hospitalized for COVID-19 was about three times higher than for patients who were hospitalized for acute infection or pneumonia in the pre-COVID era. Data from RCTs showed that LMWH-based thromboprophylaxis was more beneficial than no thromboprophylaxis in hospitalized patients, especially in those with acute infections. However, in patients at a low bleeding risk and with risk factors for thromboembolism or organ failure, such as increased D-dimer or increased oxygen requirements, therapeutic doses of LMWH/UFH should be considered. The use of antiplatelet drugs (such as aspirin and P2Y12 inhibitors) together with anticoagulants in COVID-19 patients who are not critically ill is not recommended because it can increase the bleeding risk [86]. Meanwhile, the NIH recommends the use of a therapeutic dose of heparin for patients with D-dimer levels above the upper limit of normal who require low-flow oxygen and who do not have an increased risk of bleeding. A prophylactic dose of heparin is recommended for patients who do not meet the criteria for receiving therapeutic heparin or who are not receiving a therapeutic dose of heparin for other reasons unless a contraindication exists. A therapeutic dose of oral anticoagulants for VTE prophylaxis or the prevention of COVID-19 progression is not recommended, except in a clinical trial. Finally, it recommends against the use of antiplatelet therapy to prevent COVID-19 progression or mortality in noncritically ill patients [79]. 

The NIH guideline does not recommend the use of anticoagulants and antiplatelets for the prevention of thromboembolism in nonhospitalized COVID-19 patients, except in a clinical trial. VTE prophylaxis after hospital discharge is not recommended unless there is another indication, including participation in a clinical trial. Interestingly, the NIH concludes that, in patients who are discharged after COVID-19-related hospitalization and who are at high risk of VTE and at low risk of bleeding, there is insufficient evidence to recommend either in favor or against continuing anticoagulation unless another indication for VTE prophylaxis exists [79].

In critically ill patients hospitalized with COVID-19, the ISTH concluded that intermediate and therapeutic doses of LMWH/UFH are not better than prophylactic doses at reducing the risk of adverse events, including mortality and thromboembolism [86]. Similarly, the NIH recommends the use of a prophylactic dose of heparin as VTE prophylaxis for adults who require ICU-level care, including those receiving high-flow oxygen, unless a contraindication exists. Furthermore, the NIH does not recommend the use of an intermediate dose (e.g., 1 mg/kg of enoxaparin once daily) or a therapeutic dose of anticoagulation for VTE prophylaxis in adults who require ICU-level care, including those receiving high-flow oxygen [79]. Two RCTs conducted on critically ill patients hospitalized with COVID-19 failed to show a benefit of intermediate doses of LMWH/UFH versus prophylactic doses, while the other two RCTs showed no benefit of therapeutic doses of LMWH/UFH versus lower doses for reducing mortality or the need for organ support. Meanwhile, for patients who are discharged from the hospital (Figure 2), outpatient therapy with prophylactic doses of DOAC (e.g., rivaroxaban) can be given for 30 days to reduce the risk of post-COVID-19 venous thromboembolic events. This recommendation was based on data showing that COVID-19 patients who were discharged from the hospital were still at a high risk of experiencing disease complications. Some patients still displayed markers of hypercoagulability (i.e., high D-dimer) and an increased inflammatory response (i.e., high CRP), which may increase the risk of thromboembolic events and death after hospitalization and during recovery. Therefore, in patients with persistent venous thromboembolic risk factors, such as IMPROVE scores ≥ 4 or 2–3 with D-dimer levels above the normal limits, and without contraindications (e.g., a high risk of bleeding, pregnancy, lactation), therapy with 10 mg of rivaroxaban per day should be considered [86].

## 7. Summary

The high risk of thrombosis after a COVID-19 diagnosis is a result of the complex interactions between the multiple determinants of coagulopathy (i.e., thrombosis) in COVID-19. This process involves many interrelated components, both intra- and extracellular components. Endothelial injury and dysfunction; the activation of mononuclear phagocytes, neutrophils, and platelets; and the involvement of a “cytokine storm” and tissue hypoxia have been reported to be important factors in the pathogenesis of thromboembolism in COVID-19. DVT, PE, AMI, and ischemic stroke are the most common clinical manifestations of venous and arterial thromboembolism found in COVID-19 patients. D-dimer, platelet counts, IPF, and vWF as well as TEG can be used to predict thrombotic events, to determine the prognosis of thromboembolism, and to measure the success of therapy. The treatment of thromboembolism in COVID-19 can be divided into therapeutic and preventive measures. Specifically, anticoagulation with heparin should be used in patients with strong evidence or suspicion of thromboembolism. Meanwhile, thromboprophylaxis with low doses of heparin is also recommended for both critical and noncritical COVID-19 patients who are hospitalized and who have a high risk of experiencing thromboembolism. Finally, DOAC can be given to COVID-19 patients who meet certain criteria at their time of discharge from the hospital to prevent the occurrence of thromboembolism.

## Data Availability

No new data were created.
